# Low and declining attack rates of imported typhoid fever in the Netherlands 1997–2014, in spite of a restricted vaccination policy

**DOI:** 10.1186/s12879-016-2059-0

**Published:** 2016-12-01

**Authors:** F. S. T. Suryapranata, M. Prins, G. J. B. Sonder

**Affiliations:** 1 Department of Infectious Diseases, Public Health Service (GGD) of Amsterdam, Nieuwe Achtergracht 100, PO Box 2200, 1000 CE Amsterdam, The Netherlands; 2 National Coordination Centre for Travellers’ Health Advice (LCR), Nieuwe Achtergracht 100, PO Box 1008, 1000 BA Amsterdam, The Netherlands; 3 Department of Internal Medicine, Division of Infectious Diseases, Tropical Medicine and AIDS, Academic Medical Centre, Meibergdreef 9, 1105 AZ Amsterdam, The Netherlands

**Keywords:** Typhoid fever, Epidemiology, Travel, Guidelines, Vaccination

## Abstract

**Background:**

Typhoid fever mainly occurs in (sub) tropical regions where sanitary conditions remain poor. In other regions it occurs mainly among returning travelers or their direct contacts. The aim of this study was to evaluate the current Dutch guidelines for typhoid vaccination.

**Method:**

Crude annual attack rates (AR) per 100,000 Dutch travelers were calculated during the period 1997 to 2014 by dividing the number of typhoid fever cases by the estimated total number of travelers to a specific country or region. Regions of exposure and possible risk factors were evaluated.

**Results:**

During the study period 607 cases of typhoid fever were reported. Most cases were imported from Asia (60%). Almost half of the cases were ethnically related to typhoid risk regions and 37% were cases visiting friends and relatives. The overall ARs for travelers to all regions declined significantly. Countries with the highest ARs were India (29 per 100,000), Indonesia (8 per 100,000), and Morocco (10 per 100,000). There was a significant decline in ARs among travelers to popular travel destinations such as Morocco, Turkey, and Indonesia. ARs among travelers to intermediate-risk areas according to the Dutch guidelines such as Latin America or Sub-Saharan Africa remained very low, despite the restricted vaccination policy for these areas compared to many other guidelines.

**Conclusion:**

The overall AR of typhoid fever among travelers returning to the Netherlands is very low and has declined in the past 20 years. The Dutch vaccination policy not to vaccinate short-term travelers to Latin-America, Sub-Saharan Africa, Thailand and Malaysia seems to be justified, because the ARs for these destinations remain very low. These results suggest that further restriction of the Dutch vaccination policy is justified.

## Background

Typhoid fever is caused by the bacteria *Salmonella enterica* subspecies *enterica* serovar Typhi (S. Typhi) and is primarily spread through the fecal-oral route. Symptoms usually develop 1–3 weeks after exposure, may be mild to severe, and include high fever, malaise, headache, constipation or diarrhea, rose-coloured spots on the chest, and enlarged spleen and liver [1, 2]. The estimated number of typhoid cases in low and middle income countries is around 20 million cases and 223,000 deaths occur worldwide every year [3–7]. The burden of disease occurs mainly in (sub) tropical regions where sanitary conditions remain poor. In other regions typhoid fever mainly occurs among returning travelers or their direct contacts [1, 2, 8]. Reliable data on the disease burden in (sub) tropical countries is not available since many hospitals lack facilities for blood culture and most patients are treated as outpatients, who are not registered [1, 9]. An earlier study found a decline in typhoid fever attack rates (ARs) among Dutch travelers to these countries, most likely caused by increasing hygienic standards since 1995 [10]. This suggests that disease burden in (sub) tropical countries is declining as well. Oral and parental vaccines against S. Typhi are available. The protection of these vaccines is around 30–50% and 60–70% respectively [11].

National guidelines for typhoid vaccination for travelers differ between countries. For example, the American guidelines [12] (CDC) do not distinguish between high and intermediate-risk regions, and recommend typhoid vaccination for travelers to almost all African, Asian, and Latin American countries, especially those visiting friends or relatives (VFRs) or those visiting smaller cities, villages, or rural areas. In contrast, the Canadian guidelines [13] (CATMAT) do distinguish between high-risk regions (South Asia) and intermediate-risk regions (Africa, most parts of Latin America and the rest of Asia). Typhoid vaccination is recommended for travelers to high-risk regions and is considered for travelers to intermediate-risk regions if there are individual-specific risk factors (e.g., children, VFRs, longer duration of travel). Finally, British guidelines [14] (NaTHNaC) distinguish high (e.g., children, long-term travelers, and VFRs) and low risk groups, as well as high (most parts of Africa, South and South-East Asia, and some countries in Latin America and the Middle East) and intermediate-risk (most countries in Latin America, the Middle East, Central Asia, Eastern Europe, and a few countries in Africa) regions. Typhoid vaccination is recommended for high-risk groups traveling to high-risk regions and considered for high-risk groups traveling to intermediate risk-regions.

**Fig. 1:**
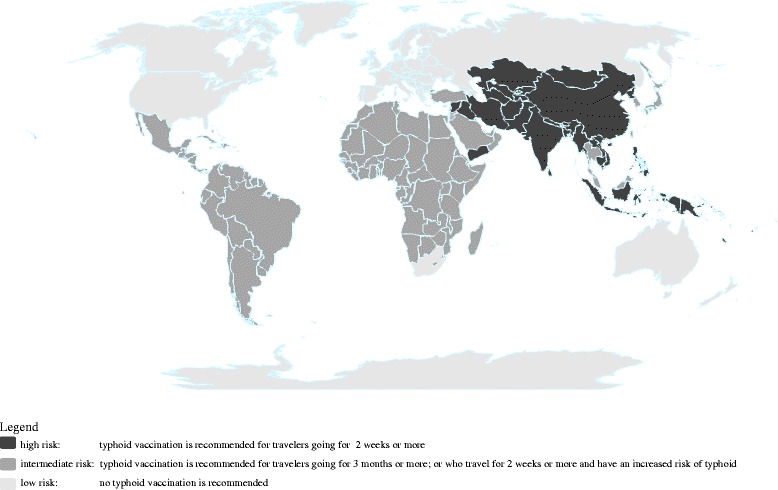
High and intermediate typhoid risk areas according to LCR guidelines

The Dutch National Coordination Centre for Travellers’ Health Advice (LCR) is responsible for the national travel medicine guidelines for the Netherlands. It is established to improve uniformity in health advice for travelers and in the quality of national vaccination centers. The LCR vaccination guidelines for typhoid are based on estimates of typhoid fever ARs among returning travelers to the Netherlands, consensus, and other national guidelines. As in the UK and Canada, LCR distinguishes high-risk and intermediate-risk regions for typhoid fever (Fig. 1) and recommends vaccination for those traveling to high-risk regions for at least 2 weeks. Travelers going to intermediate-risk regions are only recommended vaccination if they travel for at least 3 months or if they travel for at least 2 weeks and have an increased risk of typhoid fever (e.g., use antacid or have prosthetic heart valves). Compared to other national guidelines, LCR has a restricted vaccination policy, especially for travelers to Latin America and Africa.

As risks of travel-related diseases are subject to change over time, it is important to evaluate these guidelines on a regular basis. The aim of this study is to evaluate the current Dutch national guidelines for typhoid vaccination recommendations. National surveillance data on reported typhoid fever cases and travel statistics were used to retrospectively estimate trends in typhoid fever ARs among travelers by geographic region.

## Methods

### Study population and definitions

In the Netherlands, typhoid fever is a notifiable disease. A retrospective study was carried out based on all typhoid fever blood culture-confirmed cases reported by the regional Public Health Services (PHS) to the National Institute of Public Health and the Environment (RIVM) between 1997 and 2014. When a case is reported, PHS collect information on patients, including basic demographic data (age, country of birth, parents’ country of birth), date of disease onset, related cases with typhoid fever, travel history, reported vaccination status, and most likely country of infection. For our study, we categorized cases by ethnicity. If the country of birth of a case or one of the case’s parents is a high or intermediate-risk typhoid country, we classified the case’s ethnicity as from a typhoid risk region. We classified cases as “visiting friends and relatives” (VFR) if both the ethnicity of the case and the most likely country of infection was a typhoid risk region.

For this study, we defined ‘(sub) tropical countries’ as a country where hepatitis A is endemic according to WHO International Travel and Health (ITH) [15].

### Statistical analysis

Crude annual attack rates (AR) per 100,000 Dutch travelers were calculated by dividing the number of typhoid fever cases by the estimated total number of travelers to a specific country or region. For the denominator, we used data from the Continuous Holiday Survey carried out by the Dutch Tourist Board and NIPO research for the period 1997 to 2014 [16]. This survey collects travel data from a random sample of the Dutch population four times a year. Participants complete a comprehensive questionnaire related to travel and holiday destinations over the telephone. The results are weighted to represent the general Dutch population. The travel data provides only information about the destination, it does not provide information on duration of travel, purpose of travel, travel circumstances, and preventive measures taken (including vaccination).

To observe AR trends, the number of travelers each year was smoothed using a uniformly weighted moving average model: A new data series was generated by adding the current observation (n), one lagged observation (n − 1), and one forward observation (n + 1), and dividing by three. Hence, ARs were calculated for a period of three years.

Chi-square test for linear trend was performed in Epi Info version 3.03a (CDC, Atlanta, GA, USA) for analyzing time trends in AR. As country-specific travel data were not available during the whole study period for all countries, ARs were only assessed for countries where these data were available. Region-specific travel data were available for the whole study period and were used to analyze trends per region. A *p* value <0.05 was considered statistically significant.

## Results

During the study period 607 cases of typhoid fever were reported in the Netherlands. In 12 (2%) cases the country of exposure was unknown. Of all 607 cases, 89.3% (*n* = 542) were acquired in a (sub) tropical country.

**Fig. 2:**
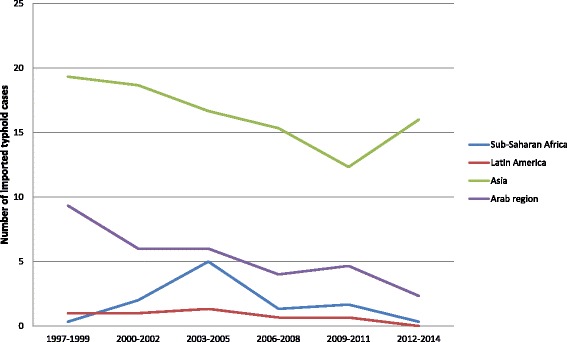
Absolute number of imported typhoid cases in the Netherlands per travel region (1997–2014)

Table 1 shows the characteristics of typhoid fever cases in the study population. The median age of the cases is 27 years (interquartile range, 15–38). Most typhoid cases (59.6%) were imported from Asia (Fig. 2). Of all 548/607 (90.3%) cases whose vaccination status was known, 118/548 (21.5%) reported to have been vaccinated against typhoid fever. Every reported case in the Netherlands is asked if they had a parental vaccination in the last three years before the first day of disease. Only those patients are considered to have been vaccinated.

**Table 1:** Characteristics of Dutch cases of typhoid fever acquired in a developing country between 1997 and 2014

Number of cases	607	
Sex		
Male	330	54.4%
Female`	277	45.6%
Median age (Interquartile range)	27	(15–38)
Ethnicity by typhoid risk region^a^		
Yes	256	42.2%
No	32	5.3%
Unknown	319	52.5%
Region of exposure		
Latin America^b^	17	2.8%
Sub-Saharan Africa^c^	37	6.1%
Arab region^d^	124	20.4%
Asia^e^	362	59.6%
Other	55	9.1%
Unknown	12	2.0%
Top 5 countries of exposure		
Indonesia	120	19.8%
India	119	19.6%
Morocco	81	13.3%
Pakistan	70	11.5%
Turkey	16	2.6%
Other	201	33.2%
VFR (Visiting Friends and Relatives)		
Yes	223	36.7%
No	64	10.5%
Unknown	320	52.7%
Severity of disease		
Hospitalization	300	49.4%
No hospitalization	97	16.0%
Hospitalization unknown	210	34.6%
Vaccination status		
Vaccinated	118	19.4%19.4%
Non vaccinated	430	70.8%
Unknown	59	9.7%

^a^Ethnicity by region: region of birth of case or one of case’s parents

^b^Latin America constitutes Central and South America, including the Caribbean

^c^Sub-Saharan Africa constitutes Western, Middle, Eastern, and Southern Africa

^d^The Arab region constitutes Northern Africa and Western Asia, including Turkey

^e^Asia constitutes Eastern and South-Eastern Asia, and the Indian subcontinent

The majority of cases with a known country of birth (*n* = 384) were born in a low-risk typhoid country (*n* = 212, 55.2%), of whom 83 (39.2%) had a parent born in a high or intermediate-risk typhoid country. Of 35/212 (16.5%) of the cases born in a low-risk typhoid country, the country of birth of both parents was unknown.

In 1997 the estimated annual number of travelers to (sub) tropical countries was 1.1 million per 15.6 million inhabitants (7.1%), increasing to 2.1 million per 16.9 million inhabitants in 2014 (12.4%) [16].

Table 2 shows the ARs for typhoid fever per 100,000 travelers to all regions and to the most popular travel destinations in (sub) tropical regions from 1997 to 2014. The overall ARs for travelers to all regions declined significantly. ARs in travelers to Latin America were low and did not significantly decline over time. ARs in travelers to Sub-Saharan Africa and to the Arab region (including Turkey) declined significantly. Among travelers to Asia, ARs were relatively high and stable over calendar time.

**Table 2:** Trends in attack rates (95% confidence interval) for typhoid fever per 100,000 Dutch travelers to (sub) tropical regions from 1997 to 2014 per region/country

	1997–1999	1998–2000	1999–2001	2000–2002	2001–2003	2002–2004	2003–2005	2004–2006	2005–2007	2006–2008	2007–2009	2008–2010	2009–2011	2010–2012	2011–2013	2012–2014	*p* value for trend
	AR (95% CI)	AR (95% CI)	AR (95% CI)	AR (95% CI)	AR (95% CI)	AR (95% CI)	AR (95% CI)	AR (95% CI)	AR (95% CI)	AR (95% CI)	AR (95% CI)	AR (95% CI)	AR (95% CI)	AR (95% CI)	AR (95% CI)	AR (95% CI)	
All regions	2,79(1,95–3,98)	2,46(1,70–3,54)	2,40(1,69–3,41)	2,03(1,40–2,94)	1,81(1,24–2,65)	1,63(1,11–2,39)	1,52(1,06–2,19)	1,30(0,89–1,90)	1,17(0,79–1,74)	1,04(0,68–1,58)	1,06(0,70–1,60)	1,02(0,67–1,55)	0,88(0,57–1,37)	0,74(0,46–1,19)	0,81(0,51–1,28)	0,87(0,55–1,36)	**0.04**
Latin America^c^	0,39(0,07–2,24)	0,40(0,07–2,27)	0,28(0,04–2,14)	0,49(0,09–2,78)	0,16(0,01–2,10)	0,26(0,03–2,00)	0,43(0,09–2,00)	0,47(0,12–1,92)	0,36(0,08–1,68)	0,17(0,02–1,33)	0,17(0,02–1,32)	0,26(0,05–1,47)	0,18(0,02–1,35)	0,09(0,01–1,27)	0,00(0,00–1,25)	0,00(0,00–1,36)	**0.22**
Netherlands Antilles	0,00(0,00–4,88)	0,00(0,00–5,91)	0,00(0,00–6,78)	0,00(0,00–9,07)	0,00(0,00–7,03)	0,00(0,00–6,03)	0,00(0,00–4,52)	0,00(0,00–4,03)	0,00(0,00–3,52)	0,00(0,00–3,59)	0,00(0,00–3,17)	0,00(0,00–3,22)	0,00(0,00–2,87)	0,00(0,00–3,22)	0,00(0,00–3,73)	0,00(0,00–4,38)	
Surinam^b^	0.0	0.0	0.0	0.0	0.0	0.0	0.0	0.0	0.0	0.0	0,00(0,00–19,87)	0,00(0,00–17,20)	0,00(0,00–15,78)	0,00(0,00–17,20)	0,00(0,00–31,14)	0,00(0,00–76,77)	
Sub-Saharan Africa^d^	0,37(0,03–4,98)	1,21(0,21–6,85)	1,61(0,35–7,53)	2,67(0,73–9,72)	5,61(2,18–14,42)∞	5,95(2,54–13,93)∞	5,17(2,21–12,11)∞	2,51(0,85–7,39)	1,29(0,32–5,24)	1,02(0,22–4,74)	1,06(0,23–4,96)	1,06(0,23–4,94)	1,17(0,29–4,76)	0,65(0,11–3,68)	0,61(0,11–3,48)	0,21(0,02–2,85)	**0.04**
Arab region^e^	2,50(1,33–4,70)	2,02(1,07–3,79)	1,62(0,86–3,05)	0,84(0,39–1,84)	0,75(0,35–1,64)	0,70(0,33–1,50)	0,56(0,26–1,22)	0,33(0,12–0,89)	0,20(0,06–0,66)	0,34(0,13–0,89)	0,49(0,23–1,07)	0,51(0,24–1,10)	0,37(0,15–0,89)	0,28(0,10–0,74)	0,23(0,08–0,66)	0,18(0,05–0,59)	**0.01**
Turkey	1,38(0,41–4,62)	0,99(0,30–3,31)	0,68(0,20–2,27)	0,21(0,04–1,17)	0,06(0,00–0,76)	0,13(0,02–0,76)	0,09(0,01–0,66)	0,09(0,01–0,67)	0,00(0,00–0,56)	0,00(0,00–0,53)	0,00(0,00–0,53)	0,00(0,00–0,52)	0,00(0,00–0,50)	0,00(0,00–0,47)	0,00(0,00–0,47)	0,00(0,00–0,46)	**0.009**
Morocco	20,63(8,31–51,24)	18,48(8,29–41,19)	22,86(10,02–52,15)	15,58(6,06–40,07)	20,00(8,05–49,67)	18,57(7,48–46,12)	15,29(6,16–37,98)	7,84(2,51–24,47)	4,96(1,48–16,62)	5,17(1,76–15,21)	6,86(2,85–16,52)	6,13(2,47–15,23)	4,74(1,77–12,68)	3,95(1,34–11,61)	3,10(0,93–10,37)	2,42(0,60–9,80)	**<0.0000001**
Asia^f^	5,38(3,46–8,37)	4,74(2,96–7,59)	5,00(3,23–7,76)	5,01(3,20–7,86)	4,30(2,65–6,98)	3,97(2,36–6,66)	3,98(2,48–6,41)	3,99(2,52–6,30)	4,53(2,91–7,05)	3,99(2,43–6,55)	4,03(2,40–6,77)	3,37(1,97–5,77)	3,00(1,73–5,20)	2,72(1,55–4,80)	3,47(2,06–5,87)^a^	4,24(2,61–6,89)^a^	**0.26**
Thailand	0,00(0,00–7,39)	0,00(0,00–7,73)	0,00(0,00–7,03)	0,00(0,00–7,07)	0,51(0,04–6,86)	0,45(0,03–6,11)	0,43(0,03–5,80)	0,00(0,00–5,19)	0,00(0,00–5,54)	0,00(0,00–5,16)	0,00(0,00–4,77)	0,00(0,00–4,18)	0,00(0,00–4,07)	0,00(0,00–4,42)	0,00(0,00–5,14)	0,00(0,00–5,65)	**0.45**
Malaysia	0,00(0,00–60,62)	0,00(0,00–15,78)	0,00(0,00–11,41)	0,00(0,00–10,48)	0,00(0,00–17,46)	2,78(0,21–37,35)	5,56(0,73–42,38)	3,03(0,40–23,12)	1,28(0,10–17,24)	0,00(0,00–12,80)	0,00(0,00–15,36)	0,00(0,00–13,56)	0,00(0,00–12,95)	0,00(0,00–13,24)	1,49(0,11–20,07)	1,75(0,13–23,59)	**0.72**
India	15,09(4,84–47,08)	15,38(4,93–47,99)	34,88(14,90–81,64)	35,71(12,78–99,76)	75,00(29,17–192,70)	27,27(9,28–80,16)	47,62(22,68–99,97)	35,85(16,76–76,66)	38,46(18,32–80,75)	28,83(12,63–65,77)	36,04(17,16–75,66)	36,70(17,48–77,04)	23,53(11,20–49,40)	20,39(9,88–42,08)	21,93(11,26–42,70)	28,72(15,28–53,97)	**0.23**
Indonesia	16,15(8,40–31,06)	16,49(7,23–37,64)	23,47(11,73–46,96)	31,11(16,55–58,46)	20,51(10,39–40,48)	16,04(7,19–35,75)	9,60(3,73–24,68)	8,15(3,05–21,78)	7,74(3,01–19,91)	5,68(2,13–15,20)	3,68(1,25–10,82)	2,39(0,71–8,01)	1,74(0,48–6,34)	1,62(0,44–5,90)	1,73(0,47–6,30)	1,56(0,39–6,35)	**<0.0000001**

^a^Nepal outbreak in 2013 among a group of Dutch travelers

∞) Cluster of family most probably acquired in a hotel in 2003 in Nigeria

^b^Confidence interval was not calculated, because the denominator was unknown until 2007–2009

^c^Latin America constitutes Central and South America, including the Caribbean

^d^Sub-Saharan Africa constitutes Western, Middle, Eastern, and Southern Africa

^e^The Arab region constitutes Northern Africa and Western Asia, including Turkey

^f^Asia constitutes Eastern and South-Eastern Asia, and the Indian subcontinent

Countries with the highest ARs were India, Indonesia and Morocco. The ARs were highest among travelers to India and there was no significant decline over time. ARs among travelers to Morocco, Turkey, and Indonesia significantly declined over time (Table 2). Pakistan was among the top 5 countries of exposure in terms of absolute number of imported cases. Unfortunately, it was not possible to calculate ARs for Pakistan, as no data were available for travelers to Pakistan. There was no data available for travelers to Surinam until 2007–2009.

Of all the cases who were VFRs and had a known vaccination status (*n* = 205), only 9 (4.4%) had been vaccinated; of all non-VFR cases with a known vaccination status (*n* = 57), 17 (29.8%) had been vaccinated (*p* < 0.001).

## Discussion

This study shows that, although the number of travelers increased, the overall attack rate of typhoid fever among returning travelers to the Netherlands is very low, despite the restricted vaccination policy compared to other countries. In India, which had the highest AR of all travel destinations the AR has not significantly declined over the study years. Previous studies also found that the Indian subcontinent has the highest attack rates among travelers [2, 3, 8]. The ARs in travelers to other countries with a high AR in 1997–1999 (Morocco, Turkey, and Indonesia) have significantly declined in recent years.

Previous Dutch colonies include Surinam, the former Netherlands Antilles, and Indonesia. Large immigrant groups from these countries and from Turkey and Morocco live in the Netherlands. These countries are popular destinations for Dutch citizens, especially for VFRs. LCR does not recommend typhoid vaccination for any individual traveling to the former Netherlands Antilles. Approximately 90,000 Dutch citizens go there each year. It is remarkable that no typhoid cases have been imported from the former Netherlands Antilles since 1997 although some national guidelines do recommend [12] or consider [13] typhoid vaccination for certain travelers to the former Netherlands Antilles.

Indonesia and India are high-risk countries according to LCR; typhoid vaccination is recommended for all travelers going to these countries for at least 2 weeks. The ARs in these two countries are among the highest of all. For Indonesia, the ARs have declined significantly. Twenty years ago, Indonesia had the highest AR of all countries, however in 2014 there were approximately 106,000 Dutch travelers going to Indonesia and only one typhoid case was reported in a returning traveler (Table 2). In a previous study it was found that increasing hygienic and sanitary conditions at travel destinations strongly contributes to a decline in ARs of fecal-orally transmitted diseases among visiting travelers [10]. Our data suggest that Indonesia might now be considered an intermediate-risk country, which is what CATMAT already does [13].

Surinam, Turkey, and Morocco have been defined as intermediate-risk countries according to LCR, which means short-term travelers are not vaccinated against typhoid. Each year on average 21,000 Dutch inhabitants travel to Surinam, 604,000 to Turkey, and 44,000 to Morocco. It is therefore remarkable that no imported typhoid cases from Surinam have been reported since 1997 and no imported cases have been reported from Turkey since 2005. For Morocco, the AR has declined significantly since 1997. As for Turkey and Surinam, several guidelines [13, 14] recommend typhoid vaccination for risk-group travelers to these countries, and some guidelines [12] recommend typhoid vaccination for all travelers, which might no longer be necessary.

The Dutch vaccination policy, which has not changed since 1997, not to vaccinate short-term travelers to Latin America, Sub-Saharan Africa, Thailand, and Malaysia seems to be justified, because the ARs for these destinations remained very low. Thailand for example has a median AR of 0 per 100,000 travelers and only one traveler returning from Thailand has been diagnosed with typhoid fever since 1997, despite an estimated average of 70,000 travelers each year. Although some national guidelines do not distinguish different risk areas within Asia [12], our results show there are large differences (e.g., between India and Thailand), justifying a country-specific policy.

Since 2012 there have been annual vaccine shortages for at least a number of months. The main reason for the worldwide shortages of vaccines since 2012 were a number of recalls by the manufacturer because the batches produced did not meet the strict quality criteria. Also, there has been an increase in travelers, a higher demand and not enough vaccine in stock. During these periods of vaccine shortages, LCR amended their guidelines so that the limited available vaccines were reserved for travelers at highest risk: vaccination was temporarily advised only for those traveling for at least 2 weeks to India, Pakistan, or Bangladesh, and for travelers going for at least 4 weeks to other high-risk areas. No vaccination was recommended for travelers to intermediate-risk areas. Despite these substantial periods of limited vaccination, we did not find any increase in typhoid cases among returning travelers since 2012. This might suggest that these temporal revised LCR guidelines for typhoid fever vaccination are sufficient.

One of the limitations of our study is that the questionnaire from the Continuous Holiday Survey is in Dutch, so only Dutch speaking citizens can participate, limiting representativeness of our study. We might have underestimated the annual number of travelers and thus overestimated ARs. Unfortunately, there are no alternative estimates available.

Another limitation of our study is that neither the travelers’ statistics [16] nor the characteristics of reported typhoid cases provide information on duration of travel, purpose of travel, travel circumstances, and preventive measures taken (vaccination data is only available for cases). Therefore it was not possible to evaluate these travel-related risk factors for typhoid fever in travelers and determine whether trends might also be explained by temporal changes in these characteristics. Vaccination is not the only important measure to prevent typhoid. Previous studies show that risk factors for typhoid fever include ‘not seeking pre-travel advice’, ‘not following food and water precautions’, ‘travel to rural areas with poor sanitation’ [17], ‘being older with a longer duration of stay’ [18], ‘a longer duration of stay in general’ [19], and ‘visiting friends and relatives’ [20]. Unfortunately, due to lack of consistent reporting of demographic characteristics of cases, the country of birth of a case or the case’s parents was unknown for the majority of cases, which means it was mostly unknown whether a case was a tourist or visiting friends or relatives.

In our study almost 20% of the typhoid cases had been vaccinated, which demonstrates that the vaccine is not always effective. A new conjugate vaccine is in development in India, which could be a good alternative for high risk travelers in the future [21, 22]. In recent years there have been a number of reported multi-year outbreaks of multi-drug resistant typhoid fever [23–26]. Vaccination against typhoid fever can also prevent infection with resistant bacteria in travelers at high risk.

Of concern is that the majority of all known VFR cases were not vaccinated. This suggests that this group is difficult to reach by travel clinics. It does not say anything about the protection of the vaccine or the risk of contracting typhoid fever.

Another limitation is that the number of cases that are treated for typhoid fever abroad is unknown. Combined with the possibility of underreporting of cases in the Netherlands, this could have led to an underestimation of the calculated ARs. However, this is unlikely to affect our trend analysis.

## Conclusions

We conclude that the overall attack rate of typhoid fever among Dutch travelers is very low and has declined in travelers to several countries in the past 20 years despite a restricted vaccination policy. Attack rates among Dutch travelers to most countries (except India) continue to decline and are so low, even in recent periods of vaccine shortage and temporal restrictions in vaccinations, that typhoid fever vaccination recommendations can be further restricted.

## Abbreviations

AR: Attack rates
CATMAT: Committee to advise on tropical medicine and travel (Canadian guidelines)
CDC: Centers for disease control and prevention (American guidelines)
ITH: WHO international travel and health
LCR: Dutch national coordination centre for travellers’ health advice (Dutch guidelines)
NaTHNac: Public Health England. national travel health network and centre (British guidelines)
PHS: Public health services
RIVM: National institute of public health and the environment
S. Typhi: Salmonella Typhi
VFR: Visiting friends or relatives
WHO: World health organization
